# Multiple cutaneous granular cell tumors involving the shoulder and foot in a child; a rare case report

**DOI:** 10.1093/jscr/rjae384

**Published:** 2024-06-03

**Authors:** Baraa Amir, Amaar Amir, Salwa Sheikh, Akram Aljahdali

**Affiliations:** Imam Abdulrahman Bin Faisal University, College of Medicine, Dammam 31441, Saudi Arabia; Imam Abdulrahman Bin Faisal University, College of Medicine, Dammam 31441, Saudi Arabia; Pathology Services, Johns Hopkins Aramco Healthcare, Dhahran 34455, Saudi Arabia; Pediatric Surgery, Johns Hopkins Aramco Healthcare, Dhahran 34455, Saudi Arabia

**Keywords:** granular cell tumors, neoplasm, soft tissue tumor, surgical excision, dermatology

## Abstract

Granular cell tumors are rare soft tissue neoplasms derived from Schwann cells and are characterized by their infiltrative, non-encapsulated nests and sheets of polygonal cells with fine eosinophilic cytoplasmic granules on histology. Herin, we report a case of a 10-year-old Saudi female who presented to the hospital with multiple asymptomatic skin lesions, the largest located on the right shoulder and left foot. Preoperative workup revealed the absence of liver metastasis, and the patient underwent complete surgical excision successfully. Histopathology revealed ill-defined proliferation of large bland cells with prominent eosinophilic granular cytoplasm and mild epithelial hyperplasia consistent with granular cell tumors. Granular cell tumors are a rare entity that represent only 0.5% of all soft tissue tumors. They have characteristic histological features and can present with both malignant and being features. Due to the rarity of this disease, further research is needed to enhance our understanding and improve recognition in future practice.

## Introduction

Granular cell tumors are soft tissue neoplasms derived from Schwann cells and are a rare disease entity. They are exemplified by their infiltrative, non-encapsulated nests and sheets of polygonal cells with copious amounts of fine eosinophilic cytoplasmic granules. They can exhibit malignant and benign features. This disease can manifest in the skin, tongue, breast, respiratory tract and gastrointestinal tract, and the recommended treatment is complete surgical excision. Of note, multiplicity of these lesions, especially in children, is a rare occurrence and the objective of this case report is to enhance awareness of such a presentation and highlight the fact that multiplicity does not reflect malignant features.

## Case presentation

**Figure 1:**
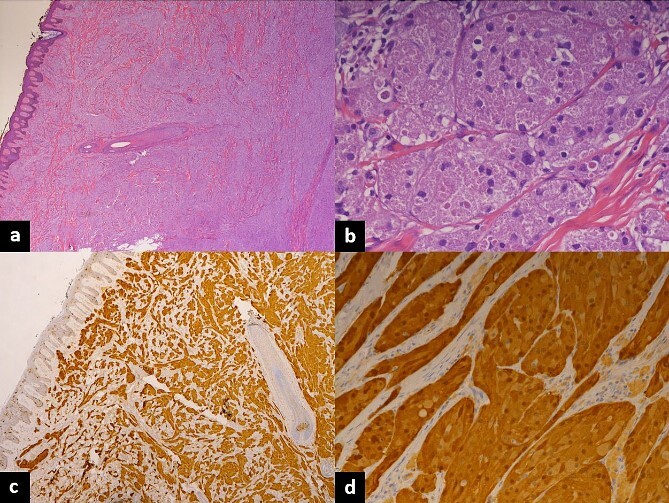
Histopathology of excised lesions. (a) Low-magnification hematoxylin and eosin stain showing ill-defined proliferation of large bland cells with mild epithelial hyperplasia. (b) High-magnification hematoxylin and eosin stain showing ill-defined proliferation of large bland cells with prominent eosinophilic granular cytoplasm. (c) Low magnification showing neoplastic cells strongly positive for S100 and negative for desmin; Ki-67 shows 2–3% of the cells to be positive. (d) High magnification showing neoplastic cells strongly positive for S100 and negative for desmin, Ki-67 shows 2–3% of the cells to be positive.

Herin, we report a case of a 10-year-old Saudi female who presented to the hospital with multiple asymptomatic skin lesions first noted at 1 year of age at the site of the BCG vaccine, then another lesion was noticed appearing on the dorsum aspect of the left foot. Four additional cutaneous lesions were noted; however, they were small and of little concern for the patient and her guardians (parents). The patient has a positive family history of balanced chromosome translocation (father) with a maternal history of habitual abortions, intrauterine fetal death with multiple congenital anomalies and a deceased sibling with multiple congenital anomalies. Her father also suffers from retroperitoneal paraganglioma. Ultrasound was preformed preoperatively to rule out liver metastasis, the liver was normal in size, parenchyma appeared homogenous and no evidence of sizable lesions was identified (unremarkable). Surgical excision was performed under general anesthesia. The skin of the right shoulder and left wound was cleaned and draped in preparation for removal. Using a scalpel, an elliptical skin incision was made around the lesion located on the right shoulder, while subcutaneous tissue dissection was done via a cauter with a needle tip. The lesion was excised completely and sent to histopathology. Granular cell tumors are usually attached to the sensory nerves; during surgical excisions, efforts should be made to preserve the nerves and sometimes shave the tumor off the nerve without damaging it, particularly on the hands and the feet. 3-0 Vicryl was used to approximate the subcutaneous tissue, and 4-0 Monocryl subcuticular suture with sterile strips were used for skin closure. Similar surgical techniques were used when removing the lesion located at the dorsum of the left foot, with the skin closed in layers using 3-0 Vicryl and 4-0 Monocryl. The patient tolerated the procedure well and was later extubated and sent to recovery on a stable condition. Specimens were received in formalin, the right shoulder lesion measured 1.5 cm, while the lesion excised from the left foot was in the form of two fragments measuring 1.0 and 0.6 cm, respectively. Microscopic sections of both regions showed ill-defined proliferation of large bland cells with prominent eosinophilic granular cytoplasm and mild epithelial hyperplasia of the overlying skin (Fig. 1a, b). Furthermore, cytological atypia, increased mitotic activity and necrosis were absent. The neoplastic cells were strongly positive for S100 and negative for desmin (Fig. 1c, d). Ki-67 shows 2–3% of the cells to be positive. These histological findings were consistent with granular cell tumors.

## Discussion

Granular cell tumor is a rare mostly soft tissue tumor with neuroectodermal differentiation. It was first described in 1854 by Weber and subsequently described in1926 by Abrikosoff as ‘Granular cell myoblastoma’, now believed to be of Schwann cell origin with vast majority of these cases behaving in a benign manner with excellent outcome, and rare malignant cases with poor prognosis reported [1–3]. These neoplasms have slow indolent growth typically 1–2 cm in diameter and may involve any anatomic location including a wide variety of sites such as peripheral soft tissue, trunk, head and neck with special predilection to tongue, and may even involve internal organs commonly esophagus. Most common sites being skin, tongue and oral cavity; in children the most common locations being oral mucosa and extremities [4]. Rare familial associations have been reported especially in Noonan and LEOPARD syndromes and 5–15% can be multiple [5–7]. It is seen in a wide age range commonly between 30 and 50 years old and only rarely in children < 5 years and elderly > 80 years old. There is slight female predominance (F:M = 5:40 with predilection for African Americans.

Clinically the skin lesion presents as papulonodular dermal or subcutaneous lesion often diagnosed as neurofibroma, dermatofibroma/fibrous histiocytoma, benign and malignant soft tissue tumors, and appendageal tumors among others. The diagnosis requires histologic examination that shows ill-defined lesion with sheets of neoplastic infiltrate of epithelioid cells separated by thin collagen bundles. The cells have abundant eosinophilic granular cytoplasm, central nuclei with some cytologic atypia, and are positive for S-100 and CD68. The skin overlying the neoplasm often shows pseudoepitheliomatous hyperplasia. Malignant tumors show characteristic histologic features including necrosis, spindling of tumor, increased mitotic activity, vesicular nuclei with large nucleoli and high proliferation index with a Ki-67 of 10% or higher [8]. A large size of > 5 cm and distant metastasis are considered negative prognostic predictors. However, necrosis and mitosis being the most effective histologic criteria for aggressive behavior and presence of metastasis, seen in 2.5% of cases, being the most definitive criterion for malignancy [2, 9]. A recent review of multiple cutaneous granular cell tumors in children reported a total of nine such cases presenting in children 5–13 years old, most presenting on extremities, and none showing any malignant features [10].

The prognosis of benign granular cell tumors is favorable as surgical excision is usually considered curative with very rare instances of recurrence. Malignant cases are more tedious and tumultuous to manage with very few successful treatment options available other than surgical removal. The 10-year survival rate in malignant cases is approximately 65%, with recurrence rates varying between 32 and 41%. Furthermore, those presenting with metastasis have a 0% survival rate after 5 years in comparison those without at 81% [11].

## Data Availability

The data used to support the findings of this study are included within the article.
